# *In Vitro* Glycoengineering of IgG1 and Its Effect on Fc Receptor Binding and ADCC Activity

**DOI:** 10.1371/journal.pone.0134949

**Published:** 2015-08-12

**Authors:** Marco Thomann, Tilman Schlothauer, Tetyana Dashivets, Sebastian Malik, Cecile Avenal, Patrick Bulau, Petra Rüger, Dietmar Reusch

**Affiliations:** 1 Pharma Technical Development Penzberg, Roche Diagnostics GmbH, Penzberg, Germany; 2 Biochemical and Analytical Research, Large Molecule Research, Pharma Research and Early Development (pRED), Roche Innovation Center, Penzberg, Germany; 3 Pharma Technical Development Basel, F. Hoffmann-La Roche Ltd, Basel, Switzerland; 4 Center for Integrated Protein Science Munich, Department Chemie, Technische Universität München, Garching, Germany; Universidade de São Paulo, BRAZIL

## Abstract

The importance and effect of Fc glycosylation of monoclonal antibodies with regard to biological activity is widely discussed and has been investigated in numerous studies. Fc glycosylation of monoclonal antibodies from current production systems is subject to batch-to-batch variability. If there are glycosylation changes between different batches, these changes are observed not only for one but multiple glycan species. Therefore, studying the effect of distinct Fc glycan species such as galactosylated and sialylated structures is challenging due to the lack of well-defined differences in glycan patterns of samples used. In this study, the influence of IgG1 Fc galactosylation and sialylation on its effector functions has been investigated using five different samples which were produced from one single drug substance batch by *in vitro* glycoengineering. This sample set comprises preparations with minimal and maximal galactosylation and different levels of sialylation of fully galactosylated Fc glycans. Among others, Roche developed the glycosyltransferase enzyme sialyltransferase which was used for the *in vitro* glycoengineering activities at medium scale. A variety of analytical assays, including Surface Plasmon Resonance and recently developed FcγR affinity chromatography, as well as an optimized cell-based ADCC assay were applied to investigate the effect of Fc galactosylation and sialylation on the *in vitro* FcγRI, IIa, and IIIa receptor binding and ADCC activity of IgG1. The results of our studies do not show an impact, neither positive nor negative, of sialic acid- containing Fc glycans of IgG1 on ADCC activity, FcγRI, and RIIIa receptors, but a slightly improved binding to FcγRIIa. Furthermore, we demonstrate a galactosylation-induced positive impact on the binding activity of the IgG1 to FcγRIIa and FcγRIIIa receptors and ADCC activity.

## Introduction

Glycosylation of therapeutic proteins is crucial for their biological activity as has been previously identified [1]. Glycosylation profiles vary depending on, for example, production cell type used, fermentation process, or even production scale [2, 3]. Variability in glycan patterns based on manufacturing variability was described also for marketed antibody products [4, 5]. This variability might be even more pronounced during development of monoclonal antibodies based on multiple changes implemented during process optimization.

The impact of non-fucosylated complex type Fc glycans on the effector function of monoclonal antibodies has been shown in different publications [6–9].

For galactose, the effects are controversially discussed based on different studies available. Several reports conclude that different galactosylation levels do not influence ADCC activity [10–12]. However, positive correlation between galactosylation and FcγRIIIa binding has also been observed in multiple studies [13, 14].

Terminal sialic acid has been shown to influence Fcγ receptor binding and anti-inflammatory activity [15] or antibody-dependent cellular cytotoxicity in different studies [16, 17] by reduced binding of sialylated antibody towards FcγRIIIa. However, there are also studies showing no influence of sialic acid on the FcγR interactions [18, 19].

Investigation of glycan structure-function is highly dependent on a well-defined difference between samples. Optimally, there should be variation in levels of only one glycan species (e.g. galactose) between the investigated samples, whereas the levels of all other glycan species should remain constant (e.g. afucose, mannose). This might be one reason for the contradictory results of previous studies, where samples have been used from different batches or after fractionation. In this study we started with one single batch of IgG1 and modified the glycan structures using glycoenzymes, the so-called *in vitro* glycoengineering (IVGE) approach. Using IVGE, a sample itself might still exhibit glycan heterogeneity but selective changes can be introduced, e.g. conversion from low levels to high levels of galactose.

Different groups have already employed this technique which emerged in recent years and is still under development. Different approaches are possible using specific enzymes called glycosyltransferases. One strategy is to transfer an entire glycan structure to the antibody backbone. In this case, the glycan tree has to be available as an oxazoline and the receiving protein needs to host the core N-acetyl glucosamine (GlcNAc) at the respective N-glycan site [20]. However, this technique is not very common since both oxazoline-derivatized sugars as well as specific enzymes are not easily available. Another strategy is treatment of glycan structures from their terminal ends. Cleavage of terminal glycans can easily be achieved by use of glycosidases such as sialidase or galactosidase. More difficult is the addition of terminal sugar moieties such as sialic acid or galactose. Prerequisites are the availability of activated sugars (e.g. CMP-NANA, UDP-Gal) and specific enzymes (e.g. sialyl- or galactosyltransferase)—ingredients which have not been reliably available in the past. This might be one reason why the *in vitro* glycoengineering approach was not broadly applied in the pharmaceutical industry even though this technique has been used for more than a decade for different, mostly analytical, purposes [21–25]. One advantage of *in vitro* glycoengineering is its independence from the production cell line and the production process. Thus, glycan variants of a therapeutic protein can easily be produced at milligram or gram scale for analytical assays, or even at kilogram scale for commercial application in relatively short time and with relatively low development effort.

To better understand the influence of the most common terminal sugar moieties of monoclonal antibody Fc glycans, galactose and sialic acid, on Fc-functionality, we decided to investigate this for a monoclonal IgG1 expressed in CHO cells at Roche in-house. The chosen IgG1, directed against a receptor of the EGFR family, has fully active effector function and ADCC as part of its mode of action. We applied the *in vitro* glycoengineering technique to selectively change the content of terminal galactose, and/or sialic acid of Fc glycans. A comprehensive set of analytical methods was used to confirm changes in the glycan pattern, to show molecule integrity and to study the impact of the different glycan variants on Fc-functionality of the antibody. Glycan analysis was performed using 2-amino benzamide (2-AB) labeling of released glycans with HPLC separation and fluorescence detection. Molecule integrity was checked by size exclusion and LCMS peptide mapping [26]. Several binding properties of the molecule were analyzed with state-of-the-art and newly developed characterization assays. Binding to FcγRI, IIa, and IIIa was assessed by Surface Plasmon Resonance (SPR) technology. As orthogonal method, recently developed FcγRIIa and IIIa affinity chromatography was applied. Additionally, a fully quantitative NK-cell based *in vitro* ADCC assay [27] was included in the study.

## Results

### Preparation and analysis of molecular integrity of different glycan variants

**Fig 1:**
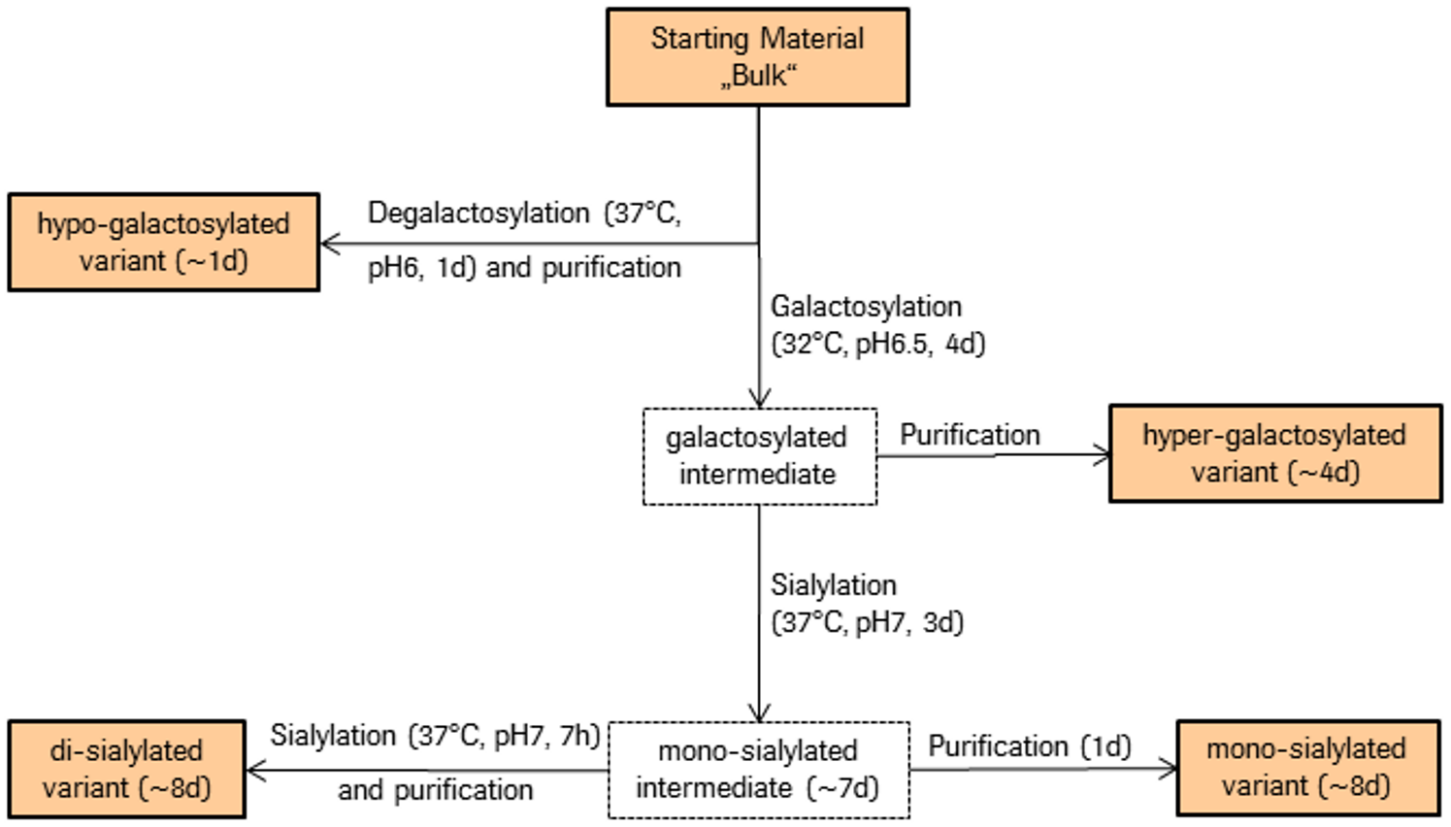
Production workflow for the different glycan variants of IgG1. Numbers in parenthesis represent the days needed for sample preparation. Starting material denoted as “bulk” is material obtained from the production process after regular fermentation and purification steps.

Starting from one single batch of IgG1, different glycan variants were produced by IVGE as described in the Materials and Methods section. Fig 1 depicts the workflow for the stepwise approach of glycan variant sample preparation. Four glycan variants were produced from the starting material: A hypo-galactosylated variant, i.e. IgG1 that comprises predominantly G0F Fc glycan species, a hyper-galactosylated variant (predominantly G2F), a mono-sialylated variant (predominantly G2S1F) and a di-sialylated variant (increased level of G2S2F) (see also Table 1 for illustration of the glycan structures). The glycosylation profile was determined by HPLC analysis after cleavage of the glycans from the protein backbone and subsequent 2-AB labeling. As shown in Table 1, the starting material (or “bulk”, material obtained from the production process after regular fermentation and purification steps) consists mainly of fucosylated glycan species without or with one single terminal galactose unit. By application of the *in vitro* glycoengineering technique about 85% purity were achieved for the hypo- and hyper-galactosylated variants without influencing levels of high-mannose or afucose (Table 1). Taking into account that some glycan species cannot be modified with the enzymes used (e.g. mannose 5), this represents the maximal possible change in galactosylation. For the mono-sialylated variant, a high level of 46.7% of G2S1F glycan species was achieved. It can be noted that the sialic acid was primarily added to the α1,3-arm using a Roche in-house human α2,6-sialyltransferase (ST6) variant (data not shown). This is in accordance with a previous publication where preferential sialylation of the α1,3-arm was observed after treatment with human ST6 [28]. For the di-sialylated variant, additional 30% of the G2S2F glycan species were added to the existing level of G2S1F.

**Table 1:** Results of *in vitro* glycoengineering.

		Sample Name				
		Bulk	hypo-galactosylated	hyper-galactosylated	mono-sialylated	di-sialylated
Relative Occurrence of Glycan Species [%]	G0F	46.5	85.3	n.d.	1.4	n.d.
	G1F	34.0	0.9	1.1	8.9	0.9
	G2F	5.0	0.4	83.7	18.6	6.1
	…	…	…	…	…	…
	G2S1F	n.d.	n.d.	n.d.	46.7	47.7
	G2S2F	n.d.	n.d.	n.d.	4.8	29.6
	M5	1.3	1.2	1.3	1.8	1.5
	Afucose	8.6	7.2	8.2	8.0	9.4
Relative Occurrence of Microheterogeneity [%]	Asn deamidation in CDR	7.0	10.7	7.8	18.2	16.1
	Iso-aspartic acid in CDR	5.2	6.5	7.1	11.2	10.5
	M255 ox	3.6	4.6	5.1	5.4	5.7
	M431 ox	1.4	2.2	1.9	2.5	2.4
Size Variant [area-%]	Monomer	99.8	99.3	98.8	97.7	98.2
	HMW	0.2	0.6	0.5	1.1	1.1
	LMW	0.0	0.0	0.8	1.2	0.7

The galactose and sialic acid content was changed significantly by *in vitro* glycoengineering. Molecule integrity was checked by LCMS peptide mapping and size exclusion chromatography. Glycan levels were determined by HPLC analysis after 2-AB labeling. Afucose levels (M5 not included) were determined by LCMS. n.d. means not detected; afucose is the sum of non-fucosylated complex or hybrid type structures; M5 is mannose five structure; GxSyF means biantennary, fucosylated glycan with x galactose and y sialic acid residues.

Potential amino acid degradation sites were monitored by LCMS tryptic peptide mapping in order to assess the impact of glycan variant sample preparation on molecular properties such as target or receptor binding. As shown in Table 1, the different sample preparation steps have a minor impact on asparagine deamidation and isoaspartic acid formation in the target binding regions. However, these observed differences in degradation are low (<10%). Molecular integrity of the samples was verified by size-exclusion chromatography as depicted in Table 1. In general, longer sample preparation time resulted in slightly reduced monomer content. As a consequence, slightly increased amounts of high- and/or low-molecular weight species were observed, resulting in a monomer content varying between 95.6% and 99.3% in the different samples.

### Fcγ receptor I, IIa, and IIIa interaction by SPR analysis

**Fig 2:**
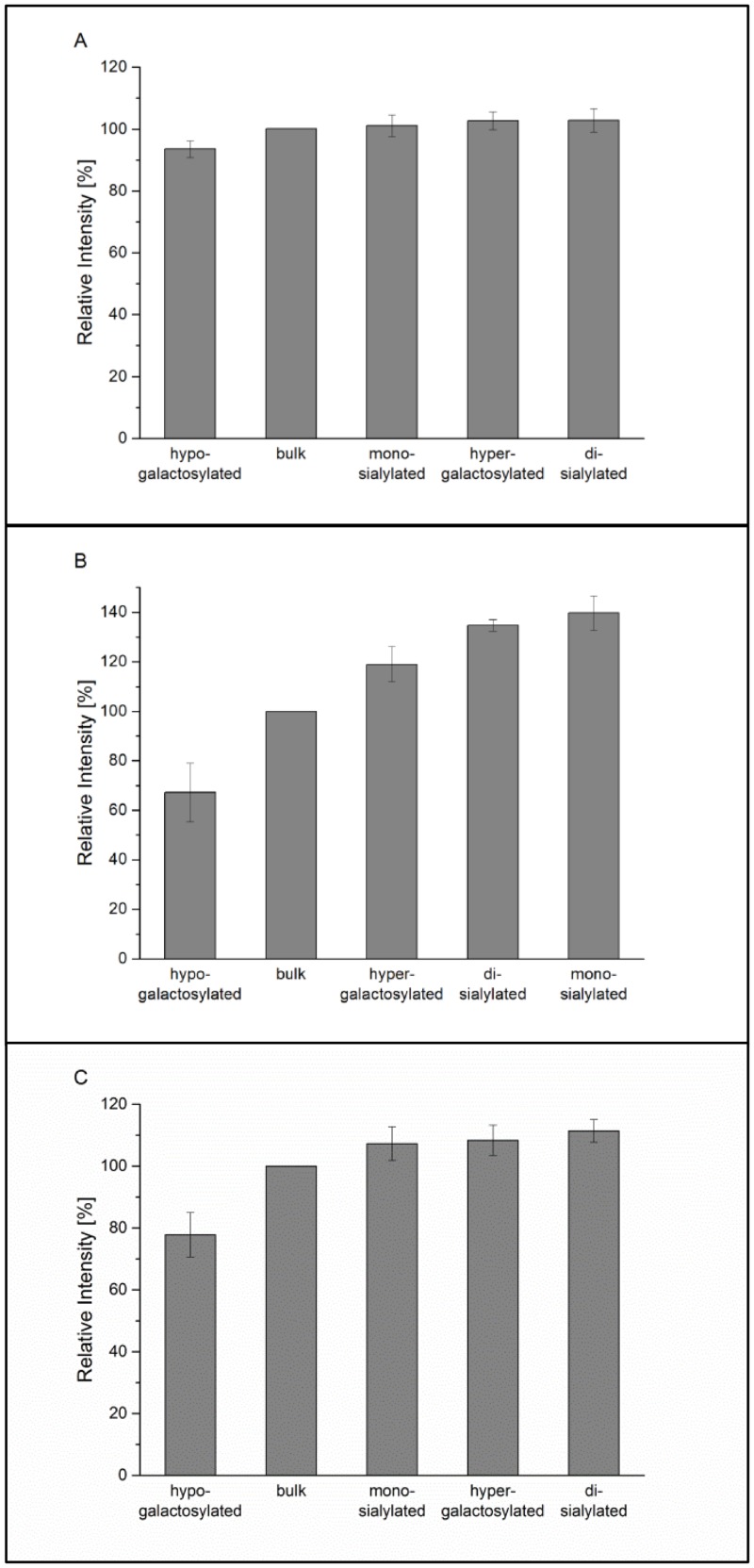
SPR analysis on FcγRI (A), FcγRIIa (B), and FcγRIIIa binding (C) in ascending intensity. Bulk material is used as reference and set to 100%. All samples are measured in triplicates.

The binding activities of the IgG1 glycovariants to the respective Fc-gamma receptors were determined by surface plasmon resonance (SPR). Here, the His-tagged Fc-gamma receptors (FcγRI, FcγRIIa_R131, and FcγRIIIa_V158) were captured via Anti-His antibody and immobilized on the chip surface. Subsequently, glycovariants in equal concentrations (300 nM) were injected as analytes. Deglycosylated IgG1 has been used as negative control in all SPR measurements. This variant failed to bind all tested FcγRs, except the highly affine FcγRI (data not shown), which is consistent with previous reports. Comparison of binding to the high affinity FcγRI showed similar interactions for all glycovariants tested (Fig 2A), whereas hypo-galactosylated material binds to FcγRIIa and FcγRIIIa with binding efficiencies between 65 and 75% (Fig 2B and 2C), compared to the bulk material. In contrast, di-galactosylated glycovariants (hyper-galactosylated, mono- and di-sialylated variants) appear to have an increased binding to FcγRIIa and FcγRIIIa (106–140%). Sialylation doesn’t seem to impair binding to FcγRs. FcγRIIa interaction even appears to be slightly improved for the sialic acid-bearing variants. Interestingly, in contrast to previously reported results [15, 17], our data do not demonstrate decreased interaction between FcγRIIIa and the sialic acid variants of Fc glycans of the IgG1.

### Fcγ receptor IIa and IIIa affinity chromatography

In addition to SPR analysis, interaction of glycovariants with the FcγRIIa and FcγRIIIa was addressed employing recently developed FcγR affinity chromatography. This analytical method is used as an orthogonal method to SPR analysis, allowing assessment of antibody receptor interaction in a qualitative manner. For these assays, human FcγRIIa_R131 or FcγRIIIa_V158 is immobilized on the column (similar to FcRn affinity chromatography, [29]). Antibody samples are loaded onto the column and eluted by a linear pH gradient from pH 8.0 to 4.0 or pH 6.0 to 3.0, respectively. Comparison of retention times enables analysis of the interaction between applied antibodies and immobilized receptors, whereas improved binding correlates with longer retention times and vice versa. In addition, FcγRIIIa affinity chromatography enables separation of fucosylated and afucosylated antibody species. The latter were shown to have up to 50-fold higher affinity to FcγRIIIa [30], which in this case, is reflected by longer retention times.

**Fig 3:**
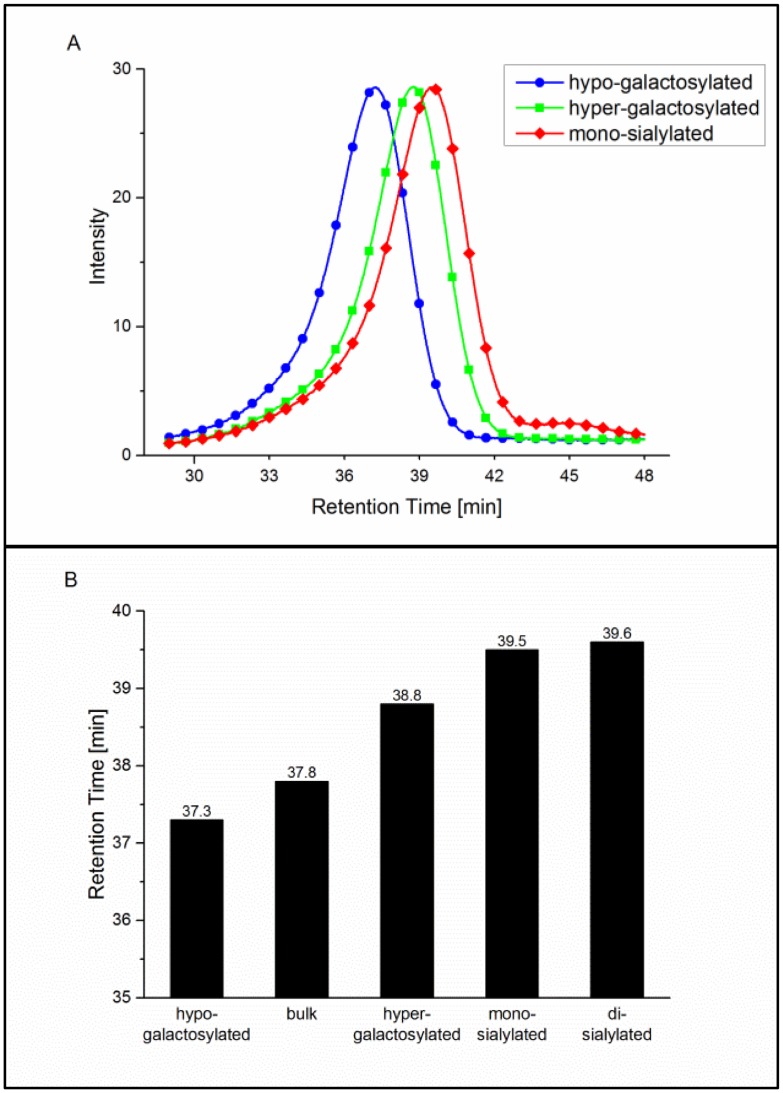
FcγRIIa column assay analysis. Normalized UV chromatograms of the hypo- and hyper-galactosylated as well as the mono-sialylated samples are exemplarily shown (A). UV absorbance was measured at 280 nm. Retention times for all batches are compared (B).

Similar to the results obtained by SPR, di-galactosylated variants show stronger interaction with the FcγRIIa on the affinity column. The effect was further enhanced by the presence of sialic acid, as demonstrated by increased retention time for these variants, compared to bulk material, whereas binding of the hypo-galactosylated variant appeared to be weaker (Fig 3). However, results from sialylated samples may only reflect a tendency since we currently have only limited experience with this assay. Deglycosylated antibody used as a negative control eluted immediately upon load, before the pH gradient was started (data not shown).

**Fig 4:**
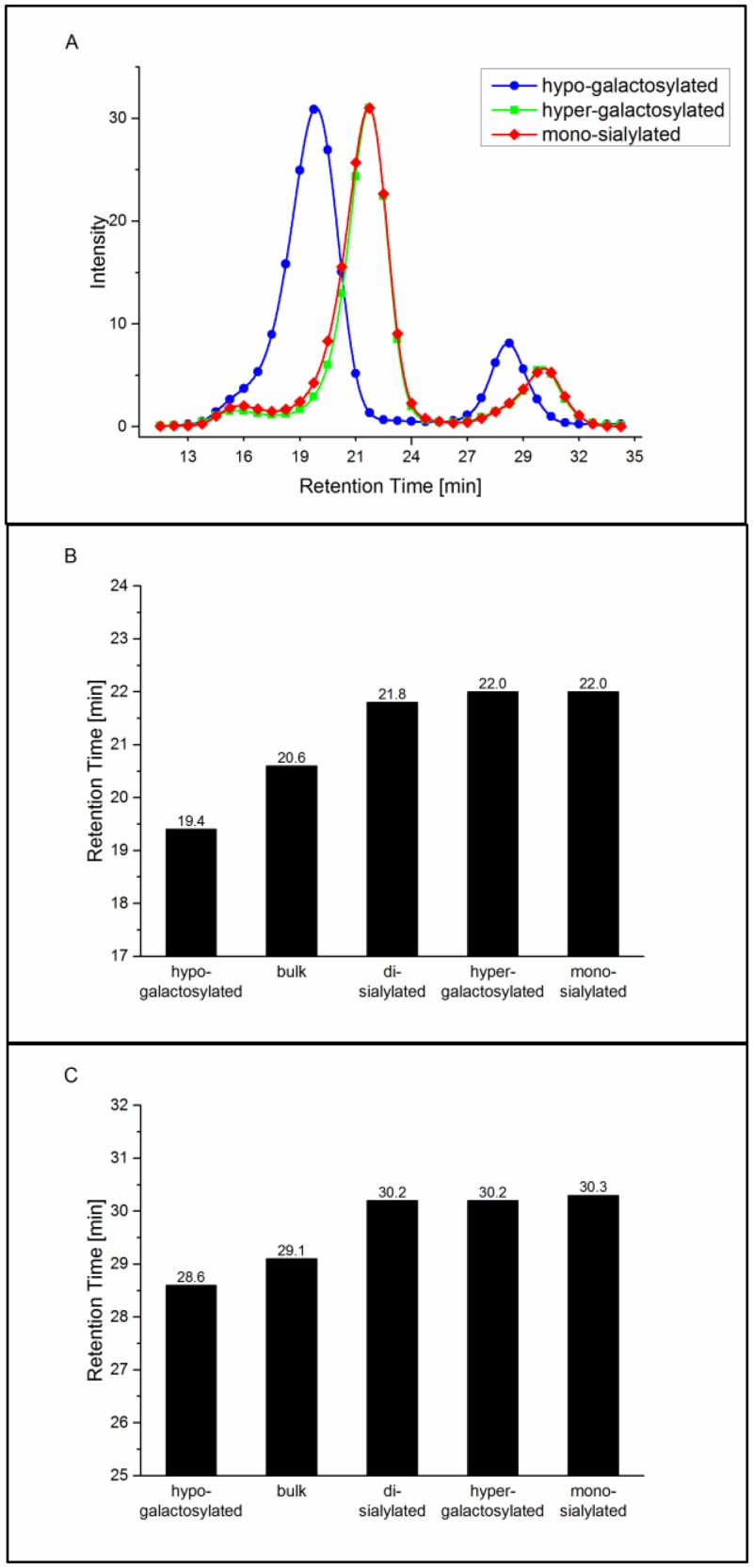
FcγRIIIa column assay analysis. Normalized UV chromatograms of the hypo- and hyper-galactosylated as well as the mono-sialylated samples are exemplarily shown (A). UV absorbance was measured at 280 nm. Retention times for all batches are compared for the fucosylated (early eluting) peak (B) and the partly/non-fucosylated (late eluting) peak (C).

As already mentioned, all tested glycan variants have a similar level of afucosylation in a range of 7–9%. Chromatographic profiles of the tested glycovariants show two peaks (Fig 4A), corresponding to two antibody species: fucosylated (main peak with retention times of 19–22 minutes) and afucosylated (small peak with retention times between 28 and 32 minutes). Comparison of the retention times of the fucosylated glycovariants demonstrated a similar binding pattern as obtained with the SPR analysis. The hypo-galacatosylated variant showed the weakest receptor interaction, revealing lower binding efficiency, compared to the bulk material (Fig 4B and 4C). Di-galactosylated variants, independent of the presence of terminal sialic acid, demonstrated a shift to longer retention times and thus stronger binding. Consistent with the reported findings, afucosylated antibodies show delayed retention times and thus improved binding affinities. Interestingly, retention patterns of afucosylated antibodies and fucosylated variants show the same tendency: Di-galactosylated antibodies show stronger binding compared to bulk material, and the opposite is true for the remaining glycovariants. These results indicate that despite the prominent effect caused by the absence of fucose, remaining sugar residues might still contribute to the antibody-receptor interaction.

The newly developed FcγRIIIa columns are a powerful tool to separate fucosylated and non-fucosylated antibodies by their binding affinity to FcγRIIIa. In addition to the effect of non-fucosylated antibodies, the FcγRIIIa column also separates the glycan variants prepared in this study with a clear correlation to the ADCC and SPR data.

## ADCC assay

**Fig 5:**
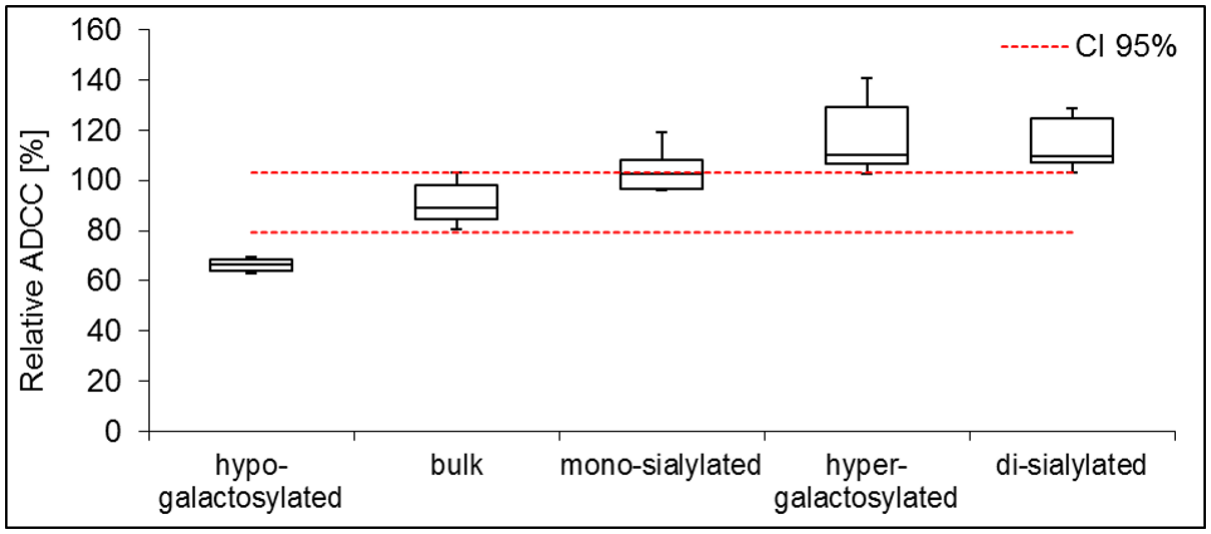
Effect of Fc glycoengineering on ADCC activity. The ADCC activities are quantified relative to a reference material set to 100% by full curve parallel line analysis. For each sample, the box plot represents 5 independent measures of duplicates. The dashed lines indicate the 95% confidence interval (CI) of the bulk material.

ADCC activity was measured using an improved cell-based assay employing Natural Killer cell lines engineered to express the high affinity variant of FcγRIIIa (V158) [27]. This assay addressed both, binding to target cells via the CDR region and activation of FcγRIIIa-positive effector cells via the Fc region of the antibody. ADCC activities were determined relative to a reference material using full curve parallel line analysis [31]. The hypo-galactosylated variant showed a decreased relative ADCC activity of 66%. A relative ADCC activity of 118% was obtained for the hyper-galactosylated variant (Fig 5). Comparison of the bulk material to the hypo- and hyper-galactosylated variants reveals that the absence of galactose has a negative impact on ADCC whereas the presence of two galactose units has a positive effect on ADCC activity. This is in accordance with the observations made for FcγRIIIa analysis by SPR technology and by the Fcγ receptor column assay.

ADCC mediated by glycan species G2S1F (104% relative ADCC) and G2S2F (115%) is not significantly different from G2F (118%). Thus, addition of sialic acid has no impact on ADCC activity.

## Summary of results

Using the IVGE approach, five samples have been generated, differing substantially in Fc glycan composition. The impact of the different glycan variants on Fc functionality was investigated and is summarized in Table 2.

**Table 2:** Summary Table—Impact of increasing galactose / sialic acid levels of IgG1 on FcγRI, IIa, and IIIa binding and ADCC activity.

	FcγRI binding	FcγRIIa binding		FcγRIIIa binding		ADCC activity
	SPR	SPR	Column assay	SPR	Column assay	ADCC assay
Increased galactosylation	no impact	increase	slight increase	increase	increase	increase
Increased sialylation	no impact	increase	slight increase	no impact	no impact	no impact

Galactose and sialic acid have no influence on FcγRI binding.

Independent from the analytical method used (SPR or affinity chromatography), galactosylation as well as sialylation tend to increase the binding to FcγRIIa_R131.

In all three methods (SPR, column assay, and ADCC), the presence of terminal galactose increases FcγRIIIa_V158 binding and consequently ADCC activity, whereas a change in terminal sialic acid content has no influence.

## Discussion

To our knowledge, this is the first time such a glycan variant sample set was generated for a monoclonal antibody. Employing the *in vitro* glycoengineering technique, defined differences in Fc glycosylation of the samples were achieved. In the past, limited availability of glycosyltransferases such as α2,6-sialyltransferase (ST6), β1,4-galactosyltransferase (GalT), and activated sugars (UDP-Gal, GMP-NANA) might have been the main challenge for the IVGE approach. Meanwhile, activated sugars, ST6, and GalT are commercially available at Roche– α2,3-sialyltransferase (ST3) is currently in development. The IVGE approach offers new opportunities in sample preparation. However, as shown in this study, generated samples might not be completely heterogeneous as it might be achievable with engineered cell lines. Nonetheless, IVGE allows for preparation of well-defined samples in sufficient amounts in a relatively short period of time compared to cell line engineering. Furthermore, IVGE is independent from the production cell line and the production process.

The mAb glycovariants in this study were investigated with regard to Fc functionality by a broad panel of analytical assays (Table 2). The obtained results are partly in accordance with and partly in contrast to previous studies.

With regard to galactosylation, the results of our study are in agreement with those from Houde *et al*. and Kumpel *et al*. [13, 14], who also observed a positive correlation of galactose with FcγRIIIa binding and ADCC activity. Houde *et al*. [13] evaluated the effect of differential galactosylation and afucosylation of IgG1 on FcγRIIIa binding. In their studies, the level of galactose was varied while keeping the afucose level constant. In those experiments, galactosylation has, next to afucosylation, a clear impact on FcγRIIIa binding. Kumpel *et al*. [14] also observed a positive contribution of increased galactosylation on ADCC using a sample set with a difference of about 26% in glycan species G0F.

Nevertheless, our results are in contrast to those obtained by Boyd *et al*., Hodoniczky *et al*., and Shinkawa *et al*. [10–12], where no impact of galactosylation on ADCC activity was reported. A possible explanation might reside in the use of Peripheral Blood Mononuclear Cells (PMBCs), which might less reliably detect and quantify small differences induced by changes in galactose levels as compared to the cell-line-based assay used in our study. Furthermore, changes in afucose levels might have overlapped and thus might have superimposed the actual impact of galactose, especially since a less pronounced effect of galactose compared to afucose has been observed in the past [30]. Maybe also the difference in galactose levels between hypo- and hyper-galactosylated samples might have been too small to measure an effect with the functional assays used.

We did neither observe a positive nor a negative impact of sialic acid on FcγRI, IIIa_V158 binding, or FcγRIIIa_V158-based ADCC activity, and only small impact on FcγRIIa_R131 binding (Figs 2 and 5), even though we were able to change the level of sialylation by about or even more than 47%. The direct involvement of IgG sialylation to FcγRII interaction remains unsolved. The inhibitory receptor FcγRIIb is structurally, closely related to the activating receptors FcγRI-III [32]. Therefore, a differentiation to the FcγRIIb based on sialylation is very unlikely and a direct involvement in an anti-inflammatory response probably not correlated to FcγRIIb binding but more to an alternative receptor [33]. The theoretical mechanisms of an anti-inflammatory response are intensely discussed [34]. For instance, in humans, DC-SIGN positive dendritic cells or macrophages were reported to detect sialic acid-rich IgGs in IVIG preparations [35].

Boyd *et al*. [10] also observed no impact of sialylation on ADCC activity. However, the difference in sialic acid levels between the samples tested was lower than 47% in their case. In contrast, other publications describe a negative impact of increased levels of sialylation on FcγRIIIa binding and ADCC activity [15–17].

It is not always clear if glycan patterns of samples used in previous studies might have had differences in other glycan species apart from sialic acid, since complete glycan analysis has not been shown after sample preparation, such as lectin fractionation [15]. In such cases, it is not clear if levels of afucose or high-mannose, which contribute to FcγRIIIa binding and ADCC activity might also have changed and therefore might impact results and conclusions of those studies.

Scallon *et al*. [17] observed reduced ADCC activity, but no change in FcγRIIIa binding when the level of sialylation in monoclonal antibodies (mAbs) produced by SP2/0 cells was increased by up to 62%. The question arose whether the location of the sialic acid residue could be of significance (α1,3 arm vs. α1,6 arm). Furthermore, SP2/0 cells produce N-glycolyl-neuraminic acid (NGNA) instead of N-acetyl-neuraminic acid (NANA, SA) which might also have an influence on binding activity and account for the observations in their study.

Overall, in our study, we were able to produce samples with significant differences in galactose and sialic acid levels of Fc glycans of an IgG1. This was achieved using the *in vitro* glycoengineering approach, a technique which has not been used extensively in the past. We carefully monitored changes in glycosylation patterns as well as molecular integrity before and after *in vitro* glycoengineering of an individual IgG1 batch prior to functional analysis.

With regard to galactosylation, we observed an impact on Fcγ receptor binding and ADCC activity. However, this impact is of much lower magnitude than the contribution from afucose. Furthermore, our sample contained primarily di-galactosylated (G2F) Fc glycans. The effect of mono-galactosylated (G1F) glycans remains to be clarified.

Concerning sialic acid, there are still numerous questions that remain open when comparing the results of our study with previous ones, where contrary results were obtained. Those conflicting observations might need further clarification and might be subject to future investigations. For example, is there a different impact of NGNA vs. NANA, does the location of sialic acid residues play a role (α1,3 arm vs. α1,6 arm), or is there a difference between terminal α2,3- and α2,6-linked sialic acid units?

Galactosyl- as well as sialyltransferase are now reliably available at Roche Custom Biotech and can easily be applied to further investigate the impact of these Fc glycans using additional IgG antibodies in future studies.

## Materials and Methods

### Preparation of glycan variants

For a schematic sample preparation workflow, see Fig 1.

#### Hypo-galactosylated variant

For degalactosylation, 750 μl β(1–4)-Galactosidase (Prozyme, GKX-5014) was added to 150 mg IgG1 (10 mU enzyme/mg antibody) and incubated at 37°C for 24 hours. The IgG1 was then purified via Protein A chromatography. The final concentration was 4.2 mg/mL.

#### Hyper-galactosylated variant

One gram IgG1 (c = 25 mg/ml) was mixed with 157 ml reaction buffer (10 mM UDP-Gal, 20 mM MnCl_2_, 100 mM MES pH 6.5). The β(1–4)-Galactosyltransferase (G5507-25U, Sigma) was diluted with dH_2_O to a concentration of 10 U/ml. 4.6 ml galactosyltransferase was added to the sample at the start of the incubation and an additional 2.3 ml was added after 2 and 3 days. The total incubation time was 4 days at 32°C. The sample was purified by Protein A chromatography. The final concentration was 8.1 mg/mL.

#### Sialylated variants (mono-sialylated, di-sialylated; α2,6-linked N-acetyl-neuraminic acid)

To achieve mono-sialylation of 500 mg of the highly galactosylated IgG1 (c = 8.1 mg/ml), 50 mg ST6-Variant1 (Roche enzyme in development; preferentially mono-sialylated glycans are obtained) and 25 ml of an aqueous CMP-NANA solution at a concentration of 10 mg/ml were added. The sample was incubated at 37°C for 3 days before Protein A purification. The final concentration was 4.8 mg/mL.

To further increase the degree of sialylation, 100 mg of the mono-sialylated sample was mixed with 3 ml of the aqueous CMP-NANA solution and 10 mg ST6-Variant2 (Roche, Cat. No. 07012250103; preferentially di-sialylated glycans are obtained). The sample was incubated for 7 hours at 37°C with subsequent Protein A purification. The final concentration was 3.8 mg/mL.

### Glycan analysis

Two-hundred micrograms of IgG1 were subjected to a buffer exchange (ammonium formate buffer (10 mM, pH8.6), followed by incubation with 2 μl PNGase F (500,000 U/ml, BioLabs) at 45°C for 1 hour and 2-AB labeling at 65°C for 2 hours with subsequent purification (Signal 2-AB Labeling Kit, Glyko, GKK404). The labeled glycans were separated by hydrophilic interaction (BEH glycan column, 1.7 μm, 2.1 x 150 mm, Waters) liquid chromatography (45 min gradient), and the fluorescence signal was detected at 420 nm (excitation wavelength at 330 nm).

### Size exclusion chromatography

Size exclusion chromatography was performed under isocratic conditions for 40 minutes at a flow rate of 0.5 ml/min using running buffer (200 mmol/l K_3_PO_4_, 250 mmol/l KCl, pH 7.0). One-hundred fifty microliter of sample at a concentration of 1 mg/ml was injected and the UV chromatograms recorded at a wavelength of 280 nm.

### LCMS peptide mapping

#### Proteolytic pH 6 digest

For the detection and quantification of Asn deamidation, Asp isomerization, and Met oxidation at the peptide level, the samples were denatured in 0.2 M His-HCl, 8 M Gua-HCl, pH 6.0 by diluting 350 μg of mAb in a total volume of 300 μl. For reduction, 10 μl of 0.1 g/ml dithiothreitol was added followed by incubation at 50°C for 1 hour. Next, the buffer solution was exchanged to digestion buffer (0.02 M His-HCl, pH 6.0) using a NAP5-gel filtration column (GE Healthcare, Buckinghamshire, UK). Subsequently, the NAP5-eluate (500 μl) was mixed with 10 μl of a 0.25 mg/ml trypsin solution (Trypsin Proteomics grade, Roche, Penzberg, Germany) in 10 mM HCl and incubated at 37°C for 18 hours.

#### Analysis of proteolytic peptides by liquid-chromatography mass-spectrometry (LC-MS)

The tryptic peptide mixture was separated by RP-UPLC (ACQUITY, Waters, Manchester, UK) on a C18 column (BEH C18 1.7 μm 2.1 x 150 mm; Waters, Manchester, UK), and the eluate analyzed online with a Synapt G2 electrospray mass spectrometer (Waters, Manchester, UK). The mobile phases consisted of 0.1% formic acid in water (solvent A) and 0.1% formic acid in acetonitrile (solvent B). The chromatography was carried out using a gradient from 1 to 35% solvent B in 45 minutes and finally from 35 to 80% solvent B in 3 minutes using a flow rate of 300 μl/min. UV absorption was measured at a wavelength of 220 nm. 3.5 μg digested protein was applied. The UPLC system and mass spectrometer were connected by PEEK-capillary tubes. Data acquisition was controlled by MassLynx V4.1 software (Waters, Manchester, UK). Parameters for MS detection were adjusted according to general experience available from peptide analysis of recombinant antibodies.

#### Data analysis for the quantification of deamidation/isomerization/oxidation levels and the level of afucosylation

Peptides of interest were identified manually by searching their *m/z*-values within the experimental mass spectrum. For the quantification, specific ion current (SIC) chromatograms of peptides of interest were generated on the basis of their monoisotopic mass and detected charge states using GRAMS AI software (Thermo Fisher Scientific, Dreieich, Germany). Relative amounts of Asn deamidation, Asp isomerization, and Met oxidation were calculated by manual integration of modified and unmodified peptide peaks. The level of afucosylation was calculated by manual integration of afucosylated and fucosylated glycan species.

### Expression and Purification of Fcγ Receptors

Human FcγRI, FcγRIIa_R131 and FcγRIIIa_V158 receptors used in this work were expressed in-house in HEK293F for FcγRI, FcγRIIa_R131 (transient expression) and in CHO cells for FcγRIIIa_V158, respectively. The CHO DG44 cell line was stably transfected [36]. Purification of the receptor was achieved by affinity chromatography using Ni Sepharose High Performance material (GE Healthcare, Munich, Germany) followed by elution with 300 mM imidazole (Sigma, Munich, Germany) an case of FcγRI, FcγRIIa_R131 and 100 mM imidazole in case of FcγRIIIa_V158, and a size exclusion chromatography on Superdex 200 26/60 column (GE Healthcare, Munich, Germany) with PBS pH7.4 (FcγRI, FcγRIIa_R131) or 2 mM MOPS, 150 mM NaCl, 0.02% Tween 20 pH 7.0 buffer (FcγRIIIa_V158). Fcγ receptors were biotinylated using the biotinylation kit from Avidity according to the manufacturer instructions (Bulk BIRA, Avidity LLC, Denver, CO, USA) and dialyzed at 4°C over night to remove excess of biotin. The product quality was characterized by standard methods. The glycosylation pattern of FcγRI and FcγRIIa is not relevant for the antibody interaction, and was not addressed in detail. Analysis of the glycosylation pattern of FcγRIIIa was described previously [36].

### Surface plasmon resonance (SPR) for FcγRI, FcγRIIa, and FcγRIIIa binding analysis

SPR interaction analysis was performed on a Biacore T200 system (GE Healthcare). For interaction analysis of FcγRs and IgG1 glycovariants, an anti-His capturing antibody (GE Healthcare) was injected to achieve a level of 12,000 resonance units (RU). Immobilization of the capturing antibody was performed on a CM5 chip using the standard amine coupling kit (GE Healthcare) at pH 4.5. FcγRIa, FcγRIIa, and FcγRIIIa were captured at a concentration of 200 nM with a pulse of 60 seconds at a flow rate of 10 μl/min. Subsequently, IgG1 glycovariants were applied at a concentration of 300 nM and a flow rate of 30 μl/min for 60 seconds. The dissociation phase was monitored for 180 seconds. The surface was regenerated by a 60 second washing step with 10 mM Glycine, pH 1.5 at a flow rate of 30 μl/min. All experiments were carried out in HBS-N buffer (10 mM HEPES, pH 7.4, 150 mM NaCl). The Biacore T100 evaluation software 2.0.3 was used for data evaluation. For the SPR IgG—Fcγ receptor analysis the data are performed in a non-kinetic analysis mode. The interaction of the IgG samples has been monitored at one fixed concentration with an n = 3. For the evaluation a report point has been set at the end of the association phase. The values of this report point were used for the evaluation of relative differences. The determination of the relative activity is from our point of view more relevant for the IgG—FcγR interaction, because this interaction does not follow a clear 1:1 Langmuir binding model and therefore the binding differences are not adequately assessed by this kind of evaluation.

### FcγRIIa and IIIa affinity chromatography

Biotinylated human FcγRIIa_R131 or FcγRIIIa_V158 was incubated with streptavidin sepharose for 2 hours with mild shaking. The receptor derivatized sepharose was packed in a Tricorn 5/50 Column housing (inner diameter 5 mm, length 50 mm, GE Healthcare) and the affinity column was equilibrated with 20 mM Tris, 150 mM NaCl pH 8.0 (FcγRIIa) or 20 mM Sodium Citrate, 150 mM NaCl pH 6.0 (FcγRIIIa) at flow rate of 0.5 ml/min using the Äkta explorer or Dionex Summit system. The antibody samples containing 50 to 100 μg in equilibration buffer were applied to the respective column. Subsequently, columns were washed with 5 column volumes of equilibration buffer. The samples were eluted with a linear pH gradient of 15 column volumes using 20 mM Citrate, 150 mM NaCl, pH 4.0 (FcγRIIa) or pH 3.0 (FcγRIIIa), respectively. The experiments were carried out at room temperature. The elution profile was obtained by continuous measurement of the absorbance at 280 nm.

### Cell-based ADCC activity assay

#### Cells

Recombinant Natural Killer effector cells expressing human FcγRIIIa_V158 were generated and cultured as previously described [27]. Target cells were purchased from American Type Culture Collection.

#### Antibody-dependent cellular cytotoxicity (ADCC)

Target cells, expressing a receptor of the EGFR family, were labeled with an acetoxymethyl ester of a fluorescence enhancing ligand (BATDA ligand, Perkin Elmer) according to the recommendations given by the supplier. Effector cells and labeled target cells were mixed in growth medium to a ratio of 5:1 and distributed in 96-well microplates. Antibody samples were diluted in growth medium and 100 μl were added to 100 μl effector-target-cell mix. Assay plates were incubated in a humidified incubator at 37°C / 5% CO_2_ for three hours. After incubation, plates were centrifuged and 20 μl supernatants from each well were transferred to a white 96-well plate. A highly fluorescent and stable chelate was obtained by complexing the released labeling agent from the lysed target cells by adding to each well two-hundred microliters of europium solution (Perkin Elmer), followed by a short incubation on a plate shaker. Controls for spontaneous and maximum release were prepared following the instructions given by the supplier of the BATDA ligand (Perkin Elmer). Time-resolved fluorescence, correlating with the amount of lysed cells, was measured in RFU (Relative Fluorescence Units) using excitation at 345 nm; emission at 615 nm. Readings were performed on a Spectramax M5 plate reader (Molecular Devices). Specific toxicity values were calculated as follows: 100% x (specific release—spontaneous release)/(maximum release—spontaneous release). For relative ADCC calculation, % specific toxicity was plotted against antibody concentration, and relative ADCC was determined by full curve parallel line analysis [31].
